# The Prevalence of Fetal Alcohol Syndrome and Its Impact on a Child’s Classroom Performance: A Case Study of a Rural South African School

**DOI:** 10.3390/ijerph14080896

**Published:** 2017-08-09

**Authors:** Melissa Lubbe, Corné van Walbeek, Nicole Vellios

**Affiliations:** 1 School of Economics, University of Cape Town, Cape Town 7700, South Africa; lubbemelissa1@gmail.com (M.L.); cwalbeek@gmail.com (C.v.W.); 2 Southern Africa Labour and Development Research Unit, University of Cape Town, Cape Town 7700, South Africa

**Keywords:** fetal alcohol syndrome, alcohol, binge drinking, pregnancy, dop system

## Abstract

Alcohol consumption is high among farm labourers in the Western and Northern Cape of South Africa. Excessive alcohol consumption during pregnancy is common, resulting in a high prevalence of Fetal Alcohol Syndrome (FAS) among children. FAS causes intellectual and behavioural problems, which create considerable obstacles to a child’s education. The aim of this study is to provide a prevalence estimate of FAS in a rural school and to examine the effects of FAS on learners’ educational outcomes. The study was conducted at a farm school near Clanwilliam in the Western Cape of South Africa. The sample comprises 166 learners from Grades 1 to 4. Educational outcomes include class scores (Afrikaans home language and mathematics), reading ability, and classroom behaviour. A physician diagnosed FAS using a three-stage process. We find FAS prevalence of 127 per 1000 (12.7%). Children with FAS score significantly lower (at the 10% level) for home language and behaviour than children who do not have FAS. Large-scale interventions in rural areas of the Western and Northern Cape that specifically target females of child-bearing age, as well as children with FAS, are necessary.

## 1. Introduction

Fetal Alcohol Syndrome (FAS) is a birth defect caused by mothers drinking alcohol whilst pregnant. The continuum of effects of prenatal alcohol exposure is referred to as Fetal Alcohol Spectrum Disorder (FASD), which encompasses FAS (most severe), Partial FAS (PFAS), Alcohol-related Neurodevelopmental Disorder (ARND) and Alcohol-related Birth Defects (ARBD) [1]. The effects on the Central Nervous System (CNS) caused by the syndrome, which include developmental delays, hyperactivity, attention deficits, learning disabilities, intellectual deficits, and sometimes seizures, complicate the educational experience of a child [2].

Mothers in South Africa often consume alcohol during pregnancy, a phenomenon frequently attributed to the “dop system” of the Western and Northern Cape where wages of farm workers were supplemented with alcohol [3]. The name “dop” originates from the Afrikaans word for a tot of alcohol. The “dop” or “tot” system originated in the 1700s when European settlers colonised fertile land in South Africa to create an agricultural economy [4]. Wine farmers, not having enough cash to pay for labour, used surplus wine as payment. The dop system also provided a way for farmers to dispose of excess wine that was deemed unfit to drink [5]. Not only were alcoholic labourers less productive, but their offspring, many of whom had FASD conditions, were unable to perform as required. The Labour Commission of 1893 acknowledged the dominance of the farmer over workers through this system, as alcohol dependency ensured that workers were prepared to work under harsh conditions to support their habit [6]. Although the use of alcohol as payment was outlawed in 1961, the free dispensation of wine “as a gift” resulted in the ongoing application of the practice [7]. Only in 2004, when the President signed the Liquor Act of 2003, was the practice of using alcohol as an inducement to employment finally prohibited [8]. The repercussions of this system still ripple through many rural communities, where the extensive use of alcohol has become a way of life for many, especially in the form of binge drinking over weekends [9].

Although laws that segregated communities on the basis of skin colour officially ended in 1994, the town of Clanwilliam in the Cederberg (like so many South African towns and cities) remains racially divided, both geographically and socially. The Cederberg is a remote agricultural area, approximately 250 km north of Cape Town. Clanwilliam is a small town with a population of about 7500 of which about 70% are of mixed race ancestry (known in South Africa as “Coloured”), 23% are African, 6% are White, and 1% are Asian/Other [10]. The vast majority (85%) of the community identified Afrikaans as their home language [10]. Over the decade spanning 2001 to 2011, the population in the Cederberg grew by 26.6% [10]. However, the town’s economic growth potential is low, weakened by factors such as high unemployment, poor literacy, a low skills base, high levels of poverty, a high incidence of HIV/AIDS, and high crime levels. An anchor employer in Clanwilliam is the Rooibos factory, purchasing most of the annual crop of Rooibos tea, the main crop of the region, which it exports to over 60 countries [11]. The town’s population is heavily dependent on government grants (especially the Child Support Grant and the Old Age Pension). The Gini coefficient (indicating income inequality) of the Cederberg is the highest in the district at 0.64 [10].

In this social context, coupled with a lack of knowledge among healthcare workers and a strained healthcare system, children’s health is often neglected, resulting in FAS often going undiagnosed. The medical diagnosis of FAS outlines three main spheres of influence: microcephaly (small head and underdeveloped brain), physical and facial abnormalities, and possible CNS abnormalities, which manifest in developmental delays, hyperactivity, low intelligence, reduced attention spans, and possible seizures [12]. Every learner with FAS or FASD presents unique challenges within the classroom, including difficulties with speech and language [12]. Research focusing on adolescents and adults with FAS shows that learning plateaus prematurely, though the age at which this happens depends on the extent of damage sustained from prenatal alcohol exposure [12]. As concrete thinkers, children with FAS find abstraction difficult [12]. Their inability to generalise from one situation to another causes considerable challenges in understanding concepts, such as time, space, figurative language, and cause and effect. Their inability to understand time means that learners cannot plan ahead and, thus, rely heavily on routine. Hyperactivity in learners with FAS resembles Attention Deficit Hyperactivity Disorder, for which it is often mistaken [13].

Although the dop system that was applied in the Western Cape for 300 years is no longer in place today, its devastating repercussions are still very pervasive [4]. In 2017, Popova et al. estimated that South Africa has the highest FAS prevalence in the world, at 58.5 per 1000 people. In fact, estimated FAS prevalence in South Africa is five times higher than Croatia, the country with the second highest prevalence of 11.5 per 1000 people [14]. Compared to the rest of the African region, South Africa is the only country with FAS prevalence greater than five cases per 1000 births [14].

In this case study, the prevalence of FAS in a rural school in Clanwilliam is estimated, together with the effects of FAS on educational outcomes of learners.

## 2. Study Context and Methods

### 2.1. Study Context

South African public schools are divided into five broadly equal groups (quintiles) for the allocation of financial resources, based on the poverty of the surrounding community. Quintile 5 is the “least poor” whilst quintile 1 is the “poorest” and comprises 8.6% of Western Cape Schools. The primary school sampled in this study is a quintile 1 school located in a remote farming region outside Clanwilliam.

The school offers schooling from Grade R (the year before a learner goes to Grade 1) to Grade 7. In 2015 there were 262 learners and 25 staff members. Two thirds of learners live in the boarding house. Most students come from homes where their physical and emotional wellbeing is often neglected (some are given nothing but sugar-water over the weekend), and where physical and sometimes sexual abuse is common. The boarding house offers security, supervision, structure, routine, and meals at set times. Since boarding is only offered during the week, learners are collected from the surrounding farms and Clanwilliam on Sunday evenings and transported home on Friday afternoons. The often dysfunctional home environment, lack of school preparation, and lack of parental support (often related to the functional illiteracy of the parents) results in discipline problems at school, with many learners repeating, many being progressed to a higher grade before they are ready, and a high dropout rate.

Although the dop system is outlawed, alcohol is still readily available to farmworkers. Every Friday (which is the weekly pay day), tradesmen hawk alcohol and other goods from farm to farm. Illegal homemade alcohol is also brewed and sold for a profit. Alcohol is usually consumed in the form of five-liter bottles of sweet, cheap wine. Four or more people usually consume a whole bottle, drinking from mugs, over the course of a weekend afternoon, often in view of children.

### 2.2. Methods

The first author spent a total of seven months at the primary school in 2015 to gather comprehensive insight into the effects of FAS on classroom performance from 166 learners: Grade 1 (*n* = 47), Grade 2 (*n* = 32), Grade 3 (*n* = 33), and Grade 4 (*n* = 54). Data on educational outcomes was obtained by collecting existing data from teachers (class scores for Afrikaans home language and mathematics), generating new data (reading score), and observing learners in the class (classroom behaviour). The learners’ class marks for mathematics and Afrikaans home language were obtained at the end of the second term, which reflect their half-year marks. There are a number of coding schemes to observe learners’ classroom behaviour directly [15]. The Behavioural Observation of Students in School (BOSS) was selected and adapted for this study since it is sensitive to the behaviours often displayed in the foundation phase (Grades 1–3) and offers a sensitive measure to detect subtle behavioural differences [15]. Each learner was subject to two 15-min BOSS sessions conducted by the first author over a period of six months. Every 10 seconds for 15 min, the first author coded behaviour according to one of six categories: (1) active engaged time (e.g., reading); (2) passive engaged time (e.g., listening to a teacher); (3) off-task motor (e.g., leaving seat); (4) off-task verbal (e.g., humming, talking to classmates); (5) off-task passive (e.g., looking out the window); or (6) teacher-directed instruction [15]. A weighting designed by the first author was assigned to each of the different behaviours based on their desirability and influence on the rest of the class (good behaviour was weighted a high number and bad behaviour a lower number). The classroom behaviour score for each child over the 15 min period was calculated to give a measure of how well the child behaved on average.

FAS diagnosis was conducted independently by a qualified medical doctor who resides and works in the district. A number was assigned to learners during the diagnosis period to protect their identity. All data was recorded on a FAS assessment form, specifically developed based on diagnosis requirements [16]. Measuring anthropometrics and dysmorphology is common in diagnosing FAS in South Africa and globally [17,18,19]. The diagnosis occurred in three phases: screening, examination, and review of hospital records. Screening of the 166 learners from Grades 1 to 4 involved basic anthropometry (i.e., weight, height, and head circumference). Any child falling below two standard deviations of the normal value for their age was included in the examination round, which was conducted during a repeat visit by the doctor. This round involved repeated anthropometric measurements, examination for typical facial characteristics (short palpebral fissure, thin upper lip, smooth philtrum, epicanthal folds, flat midface, micrognathia, flat nasal bridge, and short upturned nose) and examination for systemic manifestations (cardiac, skeletal, renal, ocular, auditory, and neurological exams) of fetal alcohol exposure [16]. The final phase was a review of maternity, clinic, and hospital notes (to which the doctor was granted access) on the mother and child.

Descriptive statistics are provided for educational outcomes (home language score, math score, behaviour score, and reading score) and physical measurements (height, weight, and head circumference). We use multiple regression analysis to predict the value of dependent variables (home language score, math score, behaviour score, and reading score) based on the value of the independent variables (gender, grade, number of days absent from school, farm/town, boarder/non-boarder, Afrikaans as home language, and years too old for grade). The data was analysed using Stata v14.1 (StataCorp LLC, College Station TX, USA). The lower and upper bounds of the 95% confidence intervals are reported for each statistic in square brackets.

Ethical clearance was obtained from the University of Cape Town (UCT) Commerce Faculty Ethical Clearance Committee (1952L Lubbe and Van Walbeek). Permission to conduct this study was also obtained from the school and from the parents of participating learners. Only one parent refused to allow the child’s participation.

## 3. Results

A local physician tested 166 learners for FAS using three rounds of investigation: the initial screening phase resulted in 52 learners (31.3% of the full sample) as possibly having FAS, the examination round further reduced the number of learners to 31 (18.7% of the full sample), and the final round of reviewing hospital records resulted in a final diagnosis of FAS in 21 learners or 12.7% of the full sample (11 boys and 10 girls) (Table 1). Some children had already been diagnosed with FAS by another doctor. The prevalence of FAS among children from farms is 16.2%, which is substantially higher than among children from town (6.6%). On all four educational outcomes children with FAS perform worse than children who do not have FAS, although not all the results are significant. The score for Afrikaans home language of learners with FAS is 7.2 marks lower than that of learners without FAS (44.9% vs. 52.1%) (*p* = 0.060). The behaviour (BOSS) score is 4.2 marks lower for FAS learners (*p* = 0.081). The reading score for girls with FAS is 15.8 marks lower than for girls who do not have FAS (*p* = 0.080), but the effect for boys is not significant.

Children with FAS are, on average, 5.2 cm shorter (*p* = 0.027), weigh 6.3 kg less (*p* = 0.001) and have a 2.3 cm smaller head circumference (*p* = 0.000) than children without FAS.

Table 2 presents multiple regression analyses for four educational outcomes for children in Grades 1 to 4. All scores are out of 100, so the numbers can be read as marks or percentages. Other than *number of days absent* and *years too old for grade*, all explanatory variables are dummy variables taking on a value of 1 if the characteristic is present, and 0 if the characteristic is not present. For example, if a learner has FAS, the variable *Fetal Alcohol Syndrome* takes on a value of 1. The coefficient in Table 2 indicates by how many marks the learner’s score changes if that characteristic is present compared to a learner who does not have that characteristic.

Home language score (column 1) decreases by 7.1 marks if a child has FAS [−14.5, 0.3], holding other factors constant. The BOSS score (column 3), which is a measure of behaviour, is reduced by 4.0 marks if a child has FAS [−8.2, 0.2]. These results are significant at the 10% level. While math (column 2) and reading scores (column 4) are also lower for children who have FAS, these results are not significant.

Females score significantly higher than males for all outcomes except math. Although we control for grades in all regressions, we do not show the coefficients on grade for regressions 1, 2 and 4 as the level of difficulty on these tests increases as a child moves through the grades, making any interpretation of those coefficients meaningless. The behaviour score is measured consistently over all four grades. Grade 2 children score 9.6 more points on average than Grade 1 children [5.6, 13.6], Grade 3 children score 11.9 more points [7.8, 16.0], and Grade 4 children score 8.1 more points [3.8, 12.4].

Children that have repeated grades score, on average, 5.2 points less [−8.9, −1.4] on home language than children who have not repeated a grade. While scores for the other educational outcomes are also lower if children have repeated grades, the results are not significant. Being a boarder significantly decreases the math score by 6.8 marks [−12.5, −1.1] but has no significant impact on the other educational outcomes. The number of days absent, living on a farm, or having Afrikaans as a home language do not appear to affect any of the four educational outcomes.

## 4. Discussion

Our estimate of 127 per 1000 births [83.6, 187.0] is close to the upper bound compared to other published studies, surpassed only by a study published in 2017 [17]. FAS prevalence in South Africa ranges from 26.5 to 129 per 1000 people (Table 3). In 2016, Roozen et al. published a worldwide systematic literature review of FASDs. Only two studies report a higher FAS prevalence than our estimate: one in the US in 1995, which reported a prevalence of 139.2 per 1000 (27/194) [20], and one in Sweden (2010) reporting a prevalence of 295.8 per 1000 (21/71) [21].

Since the dop system has its roots in viticulture, there is a general expectation that FAS prevalence is higher in these regions. However, this study, and other published studies [22,23,24,25], suggest that the prevalence of FAS in non-viticulture communities is similar to that in viticulture farming communities.

FAS learners typically perform worse than non-FAS learners, but this effect is only significant at the 10% level for home language score and classroom behaviour. May et al. found that children with FAS perform significantly worse (*p* < 0.001) than children without FAS on verbal IQ, non-verbal IQ, behaviour, and total dysmorphology scores [28]. We expected that the impact of FAS on scholastic performance would be more pronounced. The lack of significance is possibly because many learners in the school (not only those diagnosed with FAS) come from extremely deprived and dysfunctional backgrounds. Children with maltreatment histories (e.g., sexual abuse, physical abuse, emotional abuse, exposure to intimate partner violence, and neglect) often experience impairments in both their academic performance and mental well-being [30]. The deprived environment that most of the children come from decreases the average performance to the extent that it becomes difficult to identify the even lower performance of children with FAS. Had children with FAS from this study attended well-functioning, high-performing suburban schools, the scholastic difference would have likely been greater.

Having spent significant time in the classroom environment, the main author found that FAS-affected children are oversensitive to touch and other stimulation, which is a common trait of FAS [12]. Learners with FAS would shut their eyes or block their ears when the stimulation became overwhelming. They would often shut down by engaging in a non-productive activity, such as rolling a pretend-cigarette when they were over-stimulated. Children with FAS struggle to understand figurative language. Visuospatial memory difficulties mean that learners cannot focus on the details of something they are trying to copy from the board [12].

Ideally, FAS-affected children should be removed from loud or crowded classrooms to a safe haven. Unfortunately, this is not the case. Like many other rural areas in South Africa, there is no children’s home or special needs school in Clanwilliam. As a result, children with FAS are left to struggle at schools that cannot meet their specific developmental needs, which greatly reduces their chances of becoming productive members of society. In addition, there is little help for adults struggling with alcohol dependence. Many South African government-run treatment centres for alcohol-related problems have closed, and those that are left are not adequately distributed across the country. Although the number of private treatment centres has increased, the poor cannot afford the cost of these services. Specific programmes have been developed which target pregnant women, but these programmes do not reach small towns like Clanwilliam.

Although there is an awareness of FAS by health professionals in the area of Clanwilliam (some cases of FAS had been previously diagnosed), there is little being done to address the specific needs of FAS-affected learners.

We note several limitations to this study. Firstly, the sample size is small, representing 166 learners from the same school. Secondly, since we only diagnosed FAS (and not FASD) our results are not fully comparable to other studies that diagnose the full FASD spectrum. Thirdly, there may be some measurement error. Since different teachers assessed the different grades (home language and maths scores) there could be some teacher bias. There may also be desirability bias among learners (BOSS and reading scores) as learners might have improved their behaviour since they knew they were being observed. This effect is likely to be ameliorated since the first author spent a considerable amount of time in the classroom and was identified as a teacher rather than as an observer/researcher before observations commenced. There may also be some measurement error in diagnosing FAS since some hospital folders and information had been lost (in these cases the diagnosis was made on the clinical examination only). Complicated cases were also a burden, for example, a learner was excluded despite possibly having FAS because of birth abnormalities, prematurity, bacterial blood stream infection at birth, and meningitis, making the case too complicated for direct correlation to be drawn between cognitive impairment and FAS. Fourthly, definitive confirmation of maternal drinking could not be ascertained. Fifthly, the research design was such that changes in educational outcomes over a long period of time were not observed.

Further research could investigate whether the abilities of learners with FAS continue to diverge from those of their classmates, or whether they catch up. Since this is a small, and relatively closed, community, most children will presumably live in the same area in five years’ time.

## 5. Conclusions

We report an extremely high rate of FAS. Low educational attainment, low socio-economic status, easy access to cheap alcohol, and a culture of drinking rooted in the dop system have resulted in excessive alcohol use among the population of Clanwilliam. Alcohol policy responses in South Africa need to be strengthened to decrease alcohol consumption, especially among young people and women.

Not only are interventions needed to help FAS-affected children, but also to prevent future generations from continuing the cycle. This calls for a comprehensive prevention program to reduce excessive drinking and to initiate change, especially among women of child-bearing age, such as the prevention program that was successfully carried out at community health clinics in the Western Cape province for women with high-risk drinking behaviour [31]. The intervention was effective in helping women stop drinking, or drink less, during pregnancy, reducing the risk of FASD. Such interventions are urgently required in high-risk populations like that of Clanwillian.

## Figures and Tables

**Table 1:** Descriptive statistics of learners, Grades 1–4 in 2015.

	Total	Male	Female
FAS = 0 (no FAS)	145	83	62
FAS = 1 (with FAS)	21	11	10
Farm (FAS = 0)	88	50	38
Farm (FAS = 1)	17	9	8
Town (FAS = 0)	57	33	24
Town (FAS = 1)	4	2	2
**Mean of educational outcomes**			
Home language score (FAS = 0)	52.1	48.1	57.5
Home language score (FAS = 1)	44.9	41.4	48.7
*p-value*	***0.060***	*0.189*	*0.108*
Maths score (FAS = 0)	47.1	46.7	47.7
Maths score (FAS = 1)	44.0	41.9	46.4
*p-value*	*0.449*	*0.408*	*0.824*
BOSS score (FAS = 0)	63.2	59.9	67.6
BOSS score (FAS = 1)	59.0	56.1	62.2
*p-value*	***0.081***	*0.240*	***0.083***
Reading score (FAS = 0)	70.4	65.1	77.3
Reading score (FAS = 1)	62.7	63.6	61.5
*p-value*	*0.230*	*0.865*	***0.080***
**Physical measurements**			
Height in cm (FAS = 0)	127.5	127.4	127.7
Height in cm (FAS = 1)	122.3	123.4	121.0
*p-value*	***0.027***	*0.257*	***0.034***
Weight in kg (FAS = 0)	28.4	27.9	29.1
Weight in kg (FAS = 1)	22.1	23.3	20.8
*p-value*	***0.001***	***0.073***	***0.001***
Head circumference in cm (FAS = 0)	52.7	52.6	52.8
Head circumference in cm (FAS = 1)	50.4	50.3	50.3
*p-value*	***0.000***	***0.000***	***0.000***

Note: Values in bold and italic are significant at the 10% level.

**Table 2:** Multiple regression analysis for educational outcomes.

Variables	Home Language Score (1)	Math Score (2)	BOSS Score (3)	Reading Score (4)
Fetal Alcohol Syndrome (0 = no, 1 = yes)	−7.110 *	−2.539	−3.990 *	−6.073
	(3.746)	(3.720)	(2.120)	(6.213)
Gender (Female = 0, male = 1)	−7.507 ***	−1.058	−6.360 ***	−9.171 **
	(2.528)	(2.511)	**(1.430)**	(4.142)
Grade 2			9.620 ***	
			**(2.018)**	
Grade 3			11.904 ***	
			**(2.061)**	
Grade 4			8.116 ***	
			(2.192)	
Number of days absent	0.175	0.092	0.020	0.012
	(0.225)	(0.224)	(0.128)	(0.388)
Farm (Town = 0, farm = 1)	0.706	−0.580	−2.268	0.754
	(2.711)	(2.693)	(1.533)	(4.540)
Boarder (No = 0, Yes = 1)	−2.891	−6.833 **	2.175	1.744
	(2.902)	(2.882)	(1.642)	(4.740)
Afrikaans as home language (No = 0, Yes = 1)	0.493	−3.818	−0.499	5.660
	(4.409)	(4.379)	(2.493)	(7.155)
Years too old for grade	−5.158 ***	−2.672	−0.375	−2.130
	(1.886)	(1.873)	(1.065)	(3.064)
Constant	59.061 ***	65.606 ***	60.569 ***	56.846 ***
	(5.972)	(5.940)	(3.373)	(9.804)
Controls for grade	Yes	Yes	Shown	Yes
Observations	165	165	164	162
R-squared	0.198	0.282	0.349	0.223

Standard errors in parentheses *** *p* < 0.01, ** *p* < 0.05, * *p* < 0.1. Values in bold are significant at the 10% level.

**Table 3:** FAS prevalence rates from studies in South Africa.

Author/s	Year Published	Area	Viticulture Area	Grade/Age	Sample Size	FAS Per 1000
May et al. [26]	2000	Wellington, Western Cape	Yes	Grade 1	992	40.5–46.4
Viljoen et al. [22]	2003	Gauteng	No	Ages 5–10	482	26.5
Viljoen et al. [27]	2005	Wellington, Western Cape	Yes	Grade 1	857	65.2–74.2
May et al. [28]	2007	Wellington, Western Cape	Yes	Grade 1	818	67.2
Urban et al. [23]	2008	Upington & De Aar, Northern Cape	Yes	Grade 1	1835	67.2
			No			
May et al. [29]	2013	Western Cape	Yes	Grade 1	747	59.3–91
Olivier et al. [24]	2013	Aurora, Western Cape	No	Grades 1–7	160	100
Urban et al. [25]	2015	Galeshewe & Roodepan, Northern Cape	No	Grade 1	1510	55
May et al. [17]	2016	Western Cape	Yes	Grade 1	862	59–79
May [18]	2017	Western Cape	Yes	Grade 1	1083	89–129
Current study	2017	Clanwilliam, Western Cape	No	Grades 1–4	166	127
